# Prompting endogenous repair of brain injury: science fiction or reality?

**DOI:** 10.1186/s13024-022-00539-7

**Published:** 2022-05-31

**Authors:** Aurelie de Rus Jacquet, Francesca Cicchetti

**Affiliations:** 1 Centre de Recherche du CHU de Québec - Université Laval, Axe Neurosciences, Québec, QC Canada; 2 Département de Psychiatrie & Neurosciences, Université Laval, Québec, QC Canada

**Keywords:** Regenerative medicine, In situ lineage conversion, Mesial temporal lobe epilepsy with hippocampal sclerosis, Epilepsy, Neurodegenerative medicine, Gene therapy

## Abstract

Drug-refractory forms of neurological diseases could find their next breakthrough therapy in non-pharmacological approaches to brain repair. *Lentini* et al. present the potential of in situ brain regeneration to address neurodegeneration in the epileptic brain.

When millions of patients suffering from drug-refractory forms of neurological diseases face a therapeutic void, can we push the limits of current medicine to heal the brain from within? An elegant study by *Lentini *et al. [1]. provided a window into what could become tomorrow’s most effective approach; a combination of gene therapy and regenerative medicine to prompt in situ cell reprogramming [1]. This fascinating technology enables the lineage conversion of mature cells into completely different cell types at the site of injury.

Mesial temporal lobe epilepsy with hippocampal sclerosis (MTLE-HS) is a dreadful epileptic syndrome which usually manifests several years following an early childhood triggering event such as brain injury or febrile seizure [2]. The pathophysiology of this neurological disorder includes the loss of hippocampal neurons, notably GABAergic interneurons, which is accompanied by significant reactive gliosis. Patients suffering from MTLE-HS are often refractory to existing medical interventions and short-term surgery outcomes approximate 30 to 40% surgical failure. New therapeutic approaches are therefore greatly needed to target the underlying pathology and improve seizure control in this patient population. To this end, Lentini and colleagues leveraged a mouse model of MTLE-HS to demonstrate that reactive hippocampal glia can be reprogrammed in situ to regenerate functional populations of interneurons. Specifically, they propose to selectively convert proliferating glia into neurons via viral-mediated expression of Ascl1 and Dlx2, two neurogenic transcription factors (Fig. 1). The MTLE-HS model is generated by inducing kainate (KA)-mediated excitotoxicity in the dorsal hippocampus, which triggers behavioral and pathophysiological hallmarks of the disease. Instead of engineering an adeno-associated virus (AAV) as commonly described in gene delivery studies, the authors postulated that a MoMLV (Moloney murine leukemia virus)-based retroviral expression of the transgenes would be restricted to dividing cells and thus target the desired reactive glial population. *Lentini *et al. [1]. first performed a proof-of-concept experiment consisting in transplanting transduced astroglia in the dentate gyrus of the sclerotic hippocampus. They made the remarkable observation that approximately 80% of grafted Ascl1- and Dlx2-expressing cells converted into neurons, suggesting that the epileptic microenvironment did not preclude lineage conversion. To assess whether such reprogramming could be achieved in situ while being restricted to endogenous reactive glial cells, the MoMLV vector was injected in the hippocampus of MTLE-HS mice (Fig. 1). The authors showed that MoMLV transduced cells were largely non-neuronal (i.e. 71% oligodendrocytes, 14% astrocytes and 13% microglia) and glia reprogramming induced neurons with a predominantly GABAergic identity, with subpopulations depicting molecular features characteristic of interneurons at 8 weeks post-induction. Further exploration of the functional properties of these newly generated neurons revealed a striking integration within existing neuronal networks, as demonstrated by local and long-range afferent connectivity along with the re-innervation of damaged areas of the dentate gyrus. Whole-cell patch-clamp recordings confirmed that induced neurons formed GABAergic synapses evoking inhibitory postsynaptic potentials in granule cells, thus restoring connections lost/damaged in MTLE-HS. Most importantly, the investigators validated the antiepileptic potential of their cell regeneration approach by recording the electroencephalographic activity in the repopulated hippocampus. Regeneration of hippocampal interneurons and reconstruction of functional circuits reduced the number and duration of epileptic seizures by half compared to control animals, effects which correlated with the activity level of induced neurons.

**Fig. 1:**
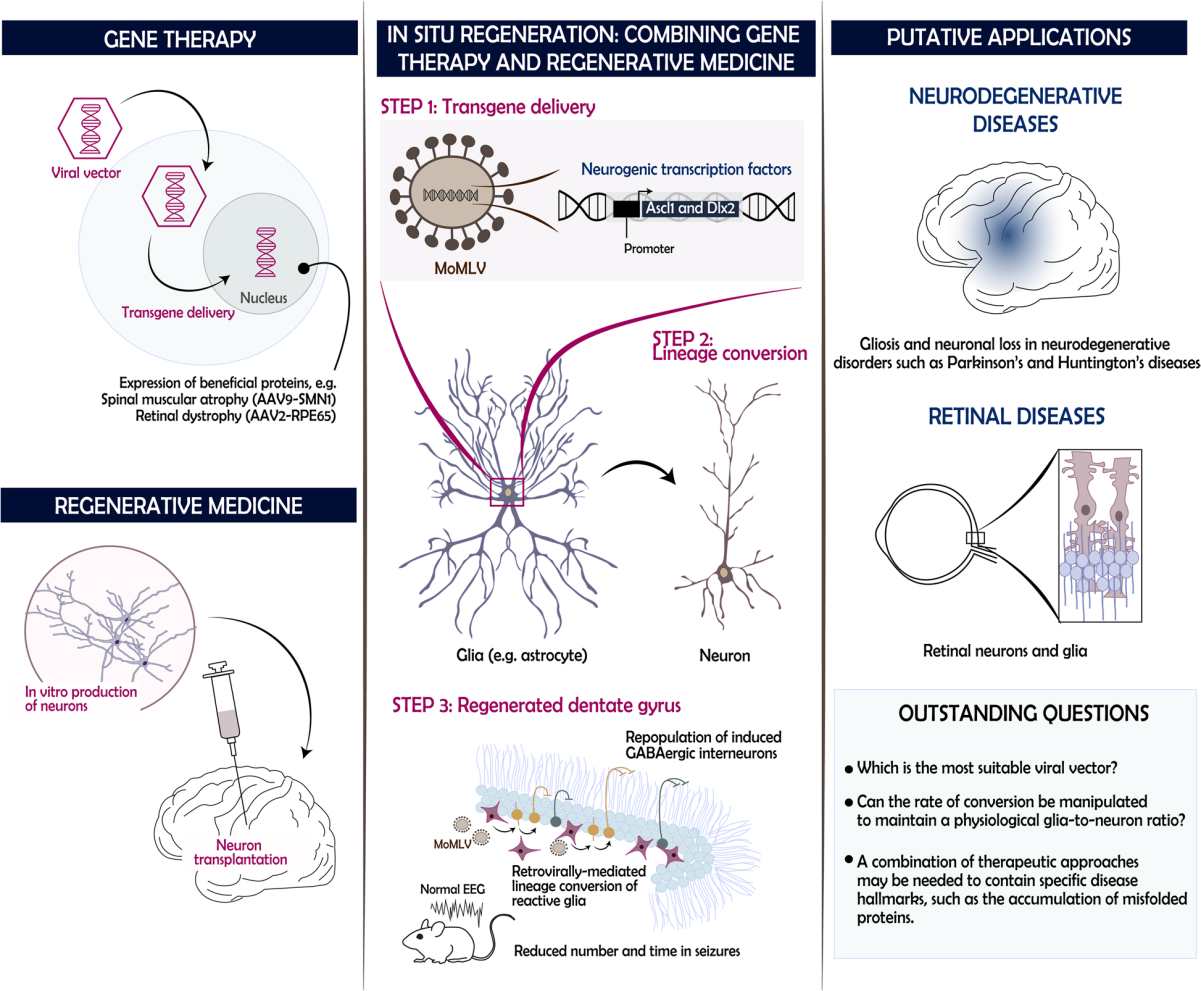
In situ cellular reprogramming. Gene therapy and regenerative medicine are two approaches under investigation to restore tissue homeostasis. The concept of in situ regeneration proposes to combine these two technologies to promote the endogenous restoration of diseased tissues. *Lentini *et al. [1]. used a MTLE-HS mouse model to study the impact of glia-to-neuron lineage conversion to restore brain function and relieve seizures. In the healthy hippocampus, GABAergic interneurons regulate the activity of granule cells, but this population of inhibitory neurons is particularly vulnerable in the epileptic brain. Reactive glial cells expressing Asl1 and Dlx2, two neurogenic transcription factors, can convert into inhibitory neurons that subsequently integrate the damaged circuit and reduce the number and length of seizures. This technology could be envisioned for several other diseases resulting from the loss of neurons and featuring extensive gliosis, regardless of the CNS foci. Abbreviations: *Ascl1* Achaete-scute homolog 1, *CNS* Central nervous system, *Dlx2* Distal-Less Homeobox 2, *EEG* Electroencephalogram, *MoMLV* Moloney murine leukemia virus

The study by *Lentini *et al. [1]. provides strong evidence that in situ cell reprogramming could attenuate refractory epileptic syndromes by replenishing lost populations of inhibitory neurons. Despite the promises of this fascinating new methodology, much work remains to be accomplished to translate these findings to the clinic. Among the key milestone to achieve, fine tuning the gene delivery method will be of paramount importance to guarantee safety (retroviruses integrate into the genome), but also ensure that the glia-to-neuron ratios remain close to those found in physiological conditions. Identifying the optimal time window to intervene will also be key to reach treatment effectiveness. The approach proposed by *Lentini *et al. [1]. relies on the presence of proliferating glial cells in the hippocampus, but it is unclear which MTLE-HS disease stage displays a sufficient number of dividing glia to provide a therapeutic benefit. The authors induced lineage conversion at an early stage of epileptogenesis (5 days post-KA), and therefore whether patients at a later stage will still benefit from this therapeutic application is unclear. Other reports have used AAV-based vectors that do not discriminate cells based on their proliferative status but on tropism towards AVV serotypes. Such studies control neurogenic gene expression using cell type-specific (e.g. GFAP) promoters. While the AAV-based approach presents with the caveat of potentially infecting non-target cells, it enabled in situ conversion of hippocampal astrocytes into GABAergic neurons and successfully rescued spontaneous recurrent seizures in a rat model of temporal lobe epilepsy [3]. Supplementary research comparing viral vectors, neurogenic genes (e.g. Ascl1, Dlx2, NeuroD1) and therapeutic windows are needed to accelerate the translation of the results to the clinic.

Gene therapies to halt the manifestation and/or progression of devastating diseases such as spinal muscular atrophy (AAV9-*SMN1*, Zolgensma®, Novartis) and biallelic RPE65 mutation-associated retinal dystrophy (AAV2-*RPE65*, Luxturna®, Spark Therapeutics) have been at the forefront of medical advances and their recent approval by regulatory institutions paves the way for the use of gene delivery-based regenerative methodologies in other settings. The targeting of reactive glia for lineage conversion is an attractive strategy because of their potentially harmful role in neurodegeneration. Their elimination by conversion into functional neurons could therefore play a dual role of removing detrimental cells while effectively correcting the loss of neurons. On a larger scale, by combining two promising technologies, in situ cellular regeneration opens new spheres of possibilities for other untreatable disorders such as Parkinson’s, Huntington’s or retinal diseases, which could very well benefit their next breakthrough therapy in lineage conversion strategies [4–6].

## Data Availability

Not Applicable.
